# One-day workshop-based training improves physical activity prescription knowledge in Latin American physicians: a pre-test post-test study

**DOI:** 10.1186/s12889-016-3883-2

**Published:** 2016-12-05

**Authors:** Maria C. Arciniegas Calle, Felipe Lobelo, Mario A. Jiménez, Diana C. Páez, Sebastian Cortés, Andrés de Lima, John Duperly

**Affiliations:** 1 Fundación Santa Fe de Bogotá, Bogotá, Colombia; 2 School of Medicine, Universidad de los Andes, Bogotá, Colombia; 3 Hubert Department of Global Health, Rollins School of Public Health, Emory University, 1599 Clifton Rd NE, Atlanta, GA USA; 4 Exercise is Medicine® Global Research and Collaboration Center, Atlanta, GA USA; 5 Department of Economics, University of Leicester, Leicester, UK

**Keywords:** Exercise, Health behavior, Counseling, Continued Education, Lifestyle Medicine

## Abstract

**Background:**

The physical inactivity pandemic and related non-communicable diseases have made it imperative for medical doctors (MDs) to effectively provide lifestyle counseling as part of prevention and treatment plans for patients. A one-day certification workshop was designed to improve MDs PA prescription knowledge, as part of the Exercise is Medicine® (EIM®) global health initiative. The objective was to determine knowledge gain of MDs participating in a standardized, one-day PA prescription workshop performed throughout Latin America (LA).

**Methods:**

A 20-question multiple-choice test on PA topics, based on international guidelines, was completed before and after the workshop. Pre and post-test analyses, without a control group, were performed on 1044 MDs after the 8-h workshop that was delivered 41 times across 12 LA countries, from January 2014 to January 2015. Knowledge improvement was determined using the class-average normalized gain and individual relative gain. T-tests with 95% confidence interval levels were conducted to analyze differences between MD specialties.

**Results:**

Test scores improved on average from 67 to 82% after the workshop (*p* <0.001). The average total individual relative gain was 29% [CI: 26 to 32%]. Relative gain by country ranged from 9.3% [CI: 2 to 16%; Nicaragua] to 73% [CI: 47 to 98%; Dominican Republic]. The mean of the 41 workshops’ class-average normalized gain was 46% [CI: 42 to 51%]. The largest groups of participants were general practitioners (GPs) (33%; *n* = 348), internal medicine (19%; *n* = 194), and family medicine (9%*n* = 92) specialists. Relative gain for GPs was not different than for all grouped primary care specialties (30% vs. 27%, *p* =0.48). The knowledge gain was higher for the workshop modules on screening/risk stratification and prescription (43% [CI: 39–48%] and 38% [CI: 34–42%], than for the module on PA benefits and risks (26% [CI: 23–28%]).

**Conclusion:**

This one-day workshop had a positive impact on the knowledge gain of MD’s on the topic of PA prescription. Although all groups of specialties increased knowledge, GPs and family medicine MDs benefited the most. This short course is an effective continuing education strategy for teaching PA assessment, counseling and prescription to MDs in Latin America, a topic rarely included in the training of MD’s in the region and the world. Further follow-up is needed to ascertain impact on PA counseling practices.

**Electronic supplementary material:**

The online version of this article (doi:10.1186/s12889-016-3883-2) contains supplementary material, which is available to authorized users.

## Background

Physical inactivity is strongly linked to the development and progression of many non-communicable chronic diseases (NCDs), with an important impact on burden of disease and life expectancy [1–3]. Physical inactivity is the fourth leading cause of global mortality, responsible for approximately 3.2 million deaths annually [4]. Overall, NCDs account for 63% of deaths globally and 80% of the NCD-related mortality occurs in low and middle-income countries [5, 6].

Conclusive evidence shows that physical activity (PA) can have comparable or increased efficacy than pharmacological therapy for the management of several NCDs [7, 8]. As the main or adjuvant therapy, PA is a cornerstone for the primary, secondary and tertiary prevention of NCDs and it also markedly reduces premature mortality caused by comorbid conditions [9–12]. This is due in part to the effect that exercise has on major shared NCD risk factors such as lowering blood pressure, inflammation, insulin resistance, triglycerides and LDL, increasing HDL, cardiovascular fitness, anti-oxidant capacity and contributing to weight loss and/or ameliorating the effects of obesity [13–19]. The global pandemic of physical inactivity and related NCDs has made it imperative for MDs and health systems to effectively provide PA and lifestyle counseling as part of prevention and treatment plan for patients.

Acting on the epidemiological evidence, many nations have agreed to improve their capacity to implement policies and programs to address NCDs and their shared behavioral origins, of which physical inactivity is one of the four pillars for action [8]. Furthermore, it is recommended that public health systems select evidence-based initiatives to ensure medical and community care personnel can effectively implement these strategies [20]. However, preventive and lifestyle medicine concepts are weak or completely lacking in many medical schools’ curricula. Therefore, most MDs lack basic knowledge, skills and abilities for the assessment, counseling and prescription of PA [21–23]. In addition, MDs and medical students’ PA habits have been found to be important predictors of their counseling practices and the relevance students give to PA counseling decreases significantly during medical school [21]; consequently, few MDs report having adequate self-efficacy to effectively prescribe PA to their patients [24].

In response to the growing inactivity epidemic, the American College of Sports Medicine (ACSM) launched in 2007 Exercise is Medicine® (EIM®), a global evidence-based initiative to routinely include PA in the treatment plan of every patient [25]. The guiding principles of EIM® emphasize the need to regularly assess PA levels in every clinical encounter, in particular for primary care providers (PCPs) and other health care professionals (HCPs). It also encourages the provision of behavioral counseling, written PA prescriptions and referrals to certified community PA resources and fitness professionals, as an integral part of the prevention and management of NCDs, and to improve the health and wellbeing of the population [25]. Since education is one of the EIM® priority areas, the Regional Center in Latin America (EIM®-RC-Latam) developed a free, one-day, certification workshop in order to improve MDs competencies to assess, counsel and prescribe PA to patients. Thus, the objective of this study was to assess knowledge gain of MDs participating in this one-day workshop as a measure of short-term impact of this educational strategy.

## Methods

Since 2011, 118 workshops have been implemented in 12 Latin American countries and around 4000 HCPs have participated [26]. A formal evaluation component was standardized starting in 2014. Overall, the workshop has been highly rated by participants, who have also reported a subjective sense of knowledge gain and many have discussed how they would modify their clinical practice in order to implement PA in their patient’s treatment plans [27].

### Study design

This is a quasi-experimental pre-test/post-test study without a control group that includes data from 41 workshops conducted in 12 Latin American countries from January 2014 to January 2015, including 1417 HCPs in a non-hospital setting. The objective of the study was to assess knowledge improvement of participants after a one-day workshop, through two objective measures of learning: a) Individual relative knowledge gain and b) class-average normalized knowledge gain. Also, we explored potential correlates of knowledge gain and differences by specialty, gender, age, country of practice, self-reported PA habits and prescription practices. The workshop included a 4-h theoretical and 2-h practical sessions.

### Surveys

At the beginning of the workshop, participants filled out a brief, previously validated survey on self-reported demographics, health status, personal PA habits (short version of the International Physical Activity Questionnaire), frequency of assessing and prescribing PA to their patients, and self-efficacy related to PA prescription [27]. Another questionnaire was administered at the end of the workshop to assess participant’s satisfaction with the course.

### Workshop intervention

The contents of the theoretical component of the workshop were based on ACSM, EIM® and other PA international guidelines and recommendations [28, 29]. Three main topics were discussed in conference-style lectures: (1) health benefits and risks associated with PA, (2) pre-participation screening and risk stratification, and (3) general principles of exercise assessment and prescription. In the practical component, participants were grouped in pairs and practiced performing a physical examination (blood pressure, resting heart rate, finger-stick glucometer), assessing anthropometric indicators (e.g. weight, height, impedance-derived fat percentage, abdominal perimeter), cardiorespiratory fitness (six-minute walk test) [30]; muscular strength (handgrip strength, sit-ups and push-ups tests); and flexibility (sit and reach test). Finally, participants completed a pre-participation risk assessment and developed a standardized PA prescription for their colleague, according to ACSM guidelines and recommendations [29].

### Pre and post-test

Participants were evaluated before and after the workshop with a 20-question multiple-choice test on basic PA topics according to the information covered in the lectures and practical sessions. Physicians scoring 80% or higher in the post-test received an international certification in exercise prescription endorsed by ACSM and EIM® RC-Latin America. The workshop was initially designed for MDs, however, participation of other HCPs (e.g. physical therapist, physical educators, nutritionists) has been allowed to foster team-based approaches and clinical-community linkages for EIM implementation. For non-MDs participants, a certificate of completion was provided [28].

### Sample

**Fig. 1:**
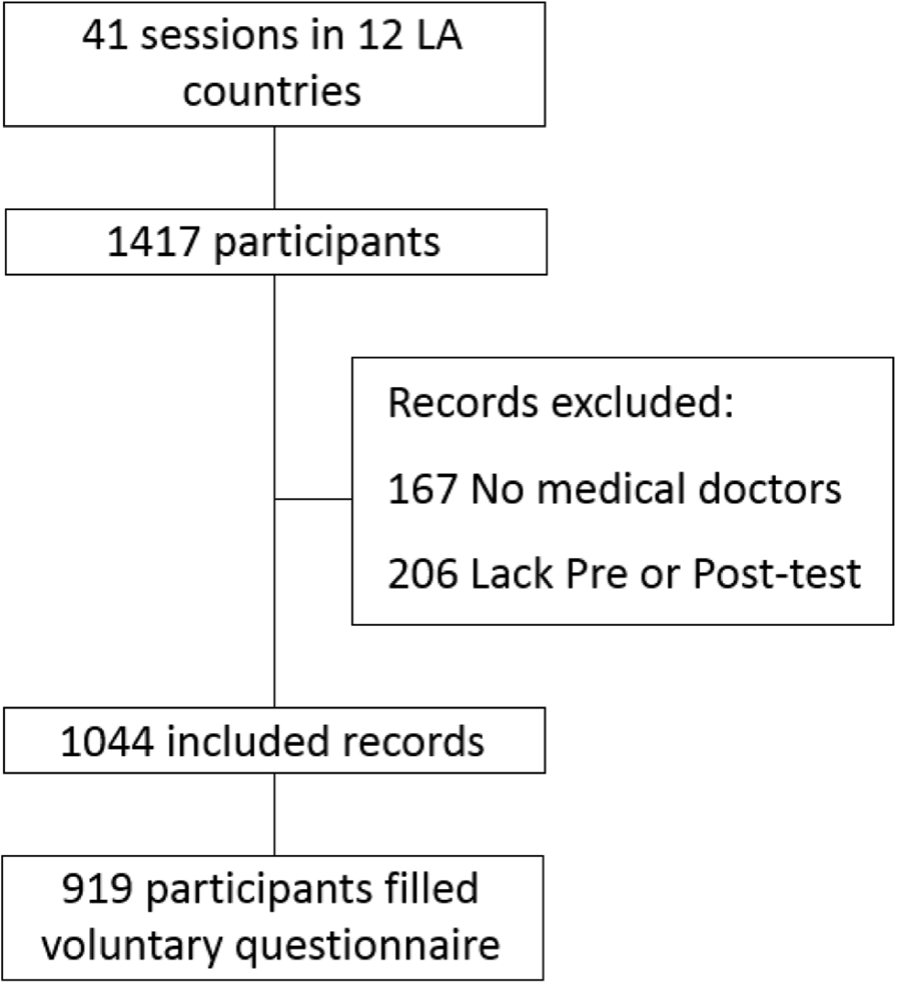
Inclusion and exclusion criteria: In order to be part of the study, participants had to have a Medical Doctor (MD) degree. Also, they had to have arrived on time and left after the post-test was administered. Participants that were not MDs were excluded. One thousand and forty-four participants met these requirements

This report focused on the impact of the workshop on physician knowledge gain; therefore data from 1044 MDs was included in this study. The only inclusion criterion was having graduated as an MD in a recognized Medical School in the region. Participants who were late for the workshop and did not take the pre-test or that left early, and thus did not complete the post-test were excluded. Participants that were not MDs or that did not correctly fill out identification information were also excluded from analyses (Fig. 1).

Recruitment of participants was done by the EIM®-RC Latin America staff and EIM national advisory boards in each country. Each country used different strategies for contacting MDs, either through e-mail databases or scientific events. Also, various medical scientific societies helped disseminate workshop information. Social media was also used as a dissemination tool. The public and private sector supported the initiative. There was a representative of the Ministry of Sports and Recreation of Colombia (COLDEPORTES) in all the workshops held in Colombia, providing participants with information on where they could refer their low-income patients for community-based PA programs. Financial support for workshop logistics (space rental, faculty travel, support personnel and workshop equipment) was provided by pharmaceutical industry partners (Astra Zeneca and Merck Sharp & Dome) in exchange for space outside of the workshop where they could advertise their relevant products. In addition, the Institute of beverages for health and wellness of the Coca Cola Company (Colombia and Mexico offices) also provided financial support for workshop logistics. The workshop was free of charge for participants. None of the sponsors of the workshop had any role in the design, evaluation, collection, analysis and interpretation of data or in the creation or delivery of the scientific material covered in the workshop. All speakers provided conflict of interest and financial support information at the beginning of the workshop. All of the participants signed an informed consent before initiation of the workshop accepting to participate in the study for evaluation purposes and all of the data collected was maintained in confidentiality. In addition, the ethics Institutional review Board of Universidad de los Andes approved the study.

### Outcome assessments

Class-average normalized knowledge gain was calculated as average actual gain/maximum possible gain (1- pre score); Individual relative knowledge gain was calculated as average actual gain (post-score minus pre-score)/pre-score and class-average normalized) [31]. The class-average normalized gain has been used in other fields of study such as engineering, mathematics and physics, as a way to measure the ratio of a whole group’s performance to the maximum achievable improvement. By using this metric, the potential confounding effect of pre-workshop baseline knowledge is attenuated thus, decreasing the need for a control group. On the other hand, the individual relative gain measures each participant’s knowledge gain in the topic [31, 32]. By using the latter, we were able to compare learning gain across different specialties to allow us to understand which groups of MDs benefitted the most and, therefore, which sub-groups should be prioritized in future training workshops.

### Statistical analysis

For descriptive data, continuous variables were expressed as mean ± standard deviations (SD), and categorical variables expressed as proportions. MDs were divided in three groups in order to explore differences between specialties and training: a) General Practitioners (GPs) in the LA context are graduated MDs with no further residency training, b) Primary Care Providers (PCPs) were specialists in family medicine, general internal medicine, obstetrics, pediatrics, and c) non-PCP specialties were sports medicine, physical medicine and other specialties such as public health/administration, surgery, dermatology, orthopedics, anesthesiology, geriatrics, pathology, genetics and internal medicine subspecialties such as immunology, endocrinology, pulmonology, nephrology, gastroenterology, hematology, intensive care specialists, and infectious diseases specialists. To assess which groups of MDs benefitted most from the workshop, the average individual relative gain was calculated for each individual specialty group. A body mass index (BMI) > 25 kg/m^2^ was classified as overweight and a BMI >30 kg/m^2^ was classified as obesity. Personal PA habits were defined as follows: meeting aerobic PA guidelines (at least 150 min/week of moderate PA, 75 min of vigorous PA or an equivalent combination), meeting resistance PA guidelines (at least 2 times/week of resistance exercises) and meeting global (both aerobic and resistance) PA guidelines. Ninety-five percent confidence intervals were obtained for all individual relative and class-average normalized gains presented. Two-tailed paired student’s t-tests with a 95% confidence interval were used to compare pre- and post-test scores for the complete sample and by each MD specialty. Also, t-tests assuming equal vs. unequal variance (according to F-tests results), with a confidence level of 95%, were conducted to compare and analyze differences in both descriptive data and individual relative gains among MD groups with the largest number of participants. Specialties with a low number of representatives were grouped into “other specialties”. Two multi-variable ordinary least squares regressions were performed taking into account the pre-test score and the individual relative gain as dependent variables to assess the potential relation with the different variables. The statistical analysis was conducted in Stata version 13 and significance set at *p* = 0.05 [33].

## Results

A total of 1044 MDs were included with the following distribution by country: Colombia (47%; *n* = 491), Mexico (16.9%; *n* = 176), Dominican Republic (10%; *n* = 104), Costa Rica (8%; *n* = 83), Argentina (3.6%; *n* = 38), Bolivia (2.8%; *n* = 29), Ecuador (2.5%; *n* = 26), Nicaragua (2.5%; *n* = 26), Uruguay (2.4%; *n* = 25), Chile (2.2%; *n* = 23), Venezuela (1.3%; *n* = 14), and Puerto Rico (0.9%; *n* = 9). The largest numbers of MDs were GPs (33%; *n* = 349), internal medicine (19%; *n* = 194), family medicine (9%; *n* = 92), sports medicine (7%; *n* = 77), cardiology (6%; *n* = 59) and pediatric specialists (5%; *n* = 52).

The general characteristics of the study population are shown in Table 1. MDs sub-groups were created in order to analyze differences among PCPs and non-PCPs as well as GPs; A total of *n* = 349 (33.4%) of participating MDs were GPs, *n* = 348 (33.3%) were PCPs and *n* = 347 (33.2%) were non-PCPs. As expected the mean age for GPs was 2 and 4 years lower than for non-PCPs and PCPs (*p* < 0.05). There was an equal representation for both genders (female: 50.3%), although there were more male GPs as well as slightly more female MDs in PCPs and non-PCPs specialties (57% vs 49% vs. 45%, respectively; *p* <0.05). Non-PCP mean BMI was lower than for GPs (25.3 vs. 26; *p* < 0.05). Overall, 53% of MDs complied with the aerobic PA recommendations and 38% with the resistance PA recommendation [28]. Non-PCPs compliance with the aerobic PA recommendation was higher than for GPs (58% vs. 53%; *p* < 0.05). In contrast, only 31% of MDs complied with both aerobic and resistance PA recommendations. Mean daily sitting time was lower for non-PCPs and PCPs than for GPs (5.2 h/day; 5.2 h/day vs. 6.4 h/day, respectively; *p* < 0.05). More than three-quarters of MDs reported having evaluated their patient’s PA levels and provided counseling, with no differences by specialty. Likewise, more than 90% of MDs agreed with the statements “MDs are responsible for promoting adequate physical activity levels” and “I will be able to provide more credible and effective counseling if I stay fit”. Agreement with the statement “I am effective in helping my patients to be physically active” was somewhat lower at 73% (Table 1).

**Table 1:** Demographics and other characteristics among MDs participating in the workshop

Demographics/Characteristics	GPs *n* = 349 (33.4%)	PCPs *n* = 348 (33.3%)	Non-PCPs *n* = 347 (33.2%)	Total *n* = 1044
Age (years)	38.2 ± 10.8 ^a^	42.8 ± 10.8 ^c^	40.5 ± 10.2 ^b^	40.5 ± 10.7
# (%) Female gender	199 (57%) ^a^	170 (49%)	156 (45%) ^b^	525 (50%)
Body mass index (kg/m^2^) # (%) Overweight and obesity (>25 kg/m^2^)	26.0 ± 4.9 152 (50%)	25.6 ± 3.8 161 (55%)	25.3 ± 3.7 ^b^ 151 (49%)	25.6 ± 4.2 464 (51%)
Aerobic PA per week [min]	202 ± 202	222 ± 211	243 ± 214	223 ± 210
Daily sitting time (hours)	6.4 ± 3.6 ^a^	5.2 ± 3.4	5.2 ± 3.4 ^b^	5.6 ± 3.5
# (%) complies with aerobic PA recommendation	149 (49%)	159 (53%)	180 (58%) ^b^	488 (53%)
# (%) complies with muscular PA recommendation	115 (38%)	112 (37%)	122 (39%)	349 (38%)
# (%) complies with global PA recommendation	90 (30%)	89 (29%)	101 (33%)	280 (31%)
# (%) evaluate PA in their clinical practice	226 (75%)	240 (80%)	243 (79%)	709 (78%)
# (%) recommends PA in their clinical practice	250 (83%)	260 (87%)	259 (84%)	769 (84%)
# (%) Agree with: “Doctors are responsible for promoting adequate physical activity levels”	294 (97%)	281 (94%)	294 (95%)	869 (95%)
# (%) Agree with: “I will be able to provide more credible and effective counseling if I stay fit”	284 (94%)	275 (91%)	288 (93%)	847 (93%)
# (%) Agree with: “I am effective in helping my patients to be physically active”	224 (74%)	212 (70%)	229 (74%)	665 (73%)

^a^Statistically significant difference between GP and PCP (*p* < 0.05)

^b^Statistically significant difference between GP and Non-PCP (*p* < 0.05)

^c^Statistically significant difference between Non-PCP and PCP (*p* < 0.05)

*GPs* General Practitioner, *PCP* primary care physicians-family medicine, general internal medicine, obstetrics, pediatrics, *Non-PCP* primary care physicians- sports medicine, physical medicine, other specialties –public health, surgery, dermatology, orthopedics, anesthesiology, geriatrics, pathology, genetics, etc.– and internal medicine subspecialties –immunology, endocrinology, pulmonology, nephrology, gastroenterology, hematology, intensive care specialists, and infectious diseases specialists

**Fig. 2:**
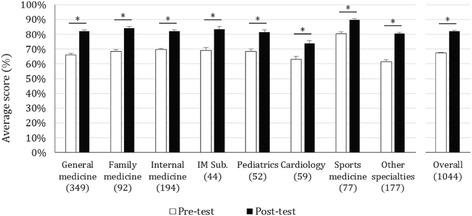
Pre and Post-test scores of participating MDs by specialty sub-groups ± standard error (*n* = 1044): The pre and post-test score was analyzed per specialty. Statistically significant differences between pre and post-test results are marked with a _* symbol (*p* < 0,001). “Other specialties” include public health, surgery, dermatology, orthopedics, anesthesiology, geriatrics, pathology, and genetics. Internal medicine subspecialties (IM Sub.) include pulmonology, nephrology, gastroenterology, and hematology. The n of each specialty is specified in parenthesis () under corresponding columns

Mean test scores for all participating MDs improved significantly from 67% (pre-test) to 82% (post-test) after the workshop (*p* < 0.001). Similarly, there was a significant increase between pre and post-test results for each specialty as shown in Fig. 2, with the highest improvement seen for general medicine (67 to 88%), family medicine (71 to 89%) and the “other specialties” (65 to 88%) sub-groups (*p* < 0.001). When analyzing the results by country, MD’s from Chile had the highest pre-test average score (80%) and accomplished a relative gain of 13%. Dominican republic on the other hand, had the lowest pre-test average score (54%) and thus had the highest relative gain at the end of the intervention (73%) (Additional file 1: Table S1).

**Fig. 3:**
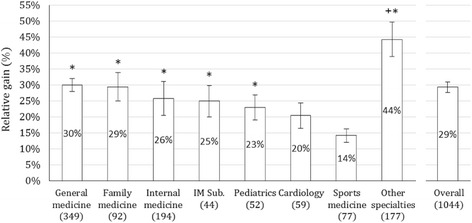
Individual Relative gain ± standard error among participating MDs by specialty sub-groups (*n* = 1044): The Individual relative gain was calculated using the formula; Average actual gain (post-score minus pre-score)/pre-score*.* The group with the greatest knowledge gain is marked with a + symbol. This gain was significantly greater than the gain exhibited by all the other specialty groups evaluated (*p* < 0.05). * Specialties marked with this symbol had a significantly higher relative knowledge gain in comparison to sports medicine (*p* < 0.05). “Other specialties” include public health, surgery, dermatology, orthopedics, anesthesiology, geriatrics, pathology, and genetics. Internal medicine subspecialties (IM Sub.) include pulmonology, nephrology, gastroenterology, and hematology. The n of each specialty is specified in parenthesis () under corresponding columns

The overall individual relative gain was calculated at 29% (CI: 26%–32%). Relative gain for GPs was no different than for PCPs (30% vs. 27%, *p* = 0.48). However, analysis by sub-groups showed that pediatrics, internal medicine, other internal medicine subspecialties, family medicine and general medicine had a significantly higher relative knowledge gain in comparison to sports medicine MDs (*p* < 0.05), (Fig. 3). In addition, the “other specialties” sub-group had the greatest relative knowledge gain (44% CI: 34–55%) and this gain was significantly greater than all the other specialty groups evaluated (*p* < 0.05) (Fig. 3). There was no significant difference in individual relative knowledge gain between PCP and non-PCP groups (*p* = 0.43).

A question-by-question analysis was completed and grouped according to the three main topics covered in the workshop. The highest individual relative knowledge gain was in the “PA prescription” module (63% CI: 57–69%), followed by the “PA benefits and risks” module (47% CI: 42–53%) and the “PA assessment” module (30% CI: 19–41%). The highest individual relative knowledge gain by specialty subgroup was in the “PA assessment” module among family medicine MDs (59% CI: 41–77%). Most of the specialties had a similar average individual relative knowledge gain by course modules, with the exception of the “other specialties” sub-group which had the highest overall average individual relative knowledge gain and the sports medicine sub-group which had the lowest, as shown in Table 2.

**Table 2:** Pre and post-test average scores and relative gain among participating MDs

Specialties	N	Benefits and Risks of PA			PA assessment			PA prescription		
		Pre	Post	Relative gain [95% CI]	Pre	Post	Relative gain [95% CI]	Pre	Post	Relative gain [95% CI]
General	349	71%	85%	26%*^+^	54%	71%	42%*	67%	88%	45%*
Practitioner				[21–30%]			[35–49%]			[37–52%]
Family	92	74%	87%	25%*	54%	73%	59%*	71%	89%	36%*
Medicine				[15–35%]			[41–77%]			[23–49%]
Internal	194	75%	85%	22%*^+^	56%	71%	46%*	73%	88%	30%^+^°
Medicine				[15–29%]			[34–57%]			[22–38%]
IM Sub.	44	74%	86%	21%^+^	55%	75%	50%*	74%	88%	24%^+^°
				[9–33%]			[28–72%]			[11–38%]
Pediatrics	52	74%	84%	19%^+^	54%	67%	37%	72%	90%	40%*
				[12–25%]			[21–52%]			[23–57%]
Cardiology	59	64%	75%	24%*^+^	56%	65%	34%	69%	80%	27%
				[15–34%]			[13–54%]			[10–44%]
Sports	77	85%	91%	11%^+^	70%	81%	21%^+^	81%	96%	20%^+^
Medicine				[6–16%]			[11–31%]			[14–26%]
Other	177	66%	83%	39%*	51%	68%	48%*	65%	88%	47%*
Specialties				[30–47%]			[37–60%]			[35–58%]
Total	1044	72%	85%	26%	55%	71%	43%	70%	89%	38%
				[23%–28%]			[39%–48%]			[34%–42%]

*: Significantly higher than Sports medicine (*p* < 0.05)

^+^: Significantly lower than other (*p* < 0.05)

°: Significantly lower than General practitioners (*p* < 0.05)

*IM Sub* Internal medicine subspecialties others than Cardiology (immunology, endocrinology, pulmonology, nephrology, gastroenterology, hematology, intensive care specialists, and infectious diseases specialists)

**Fig. 4:**
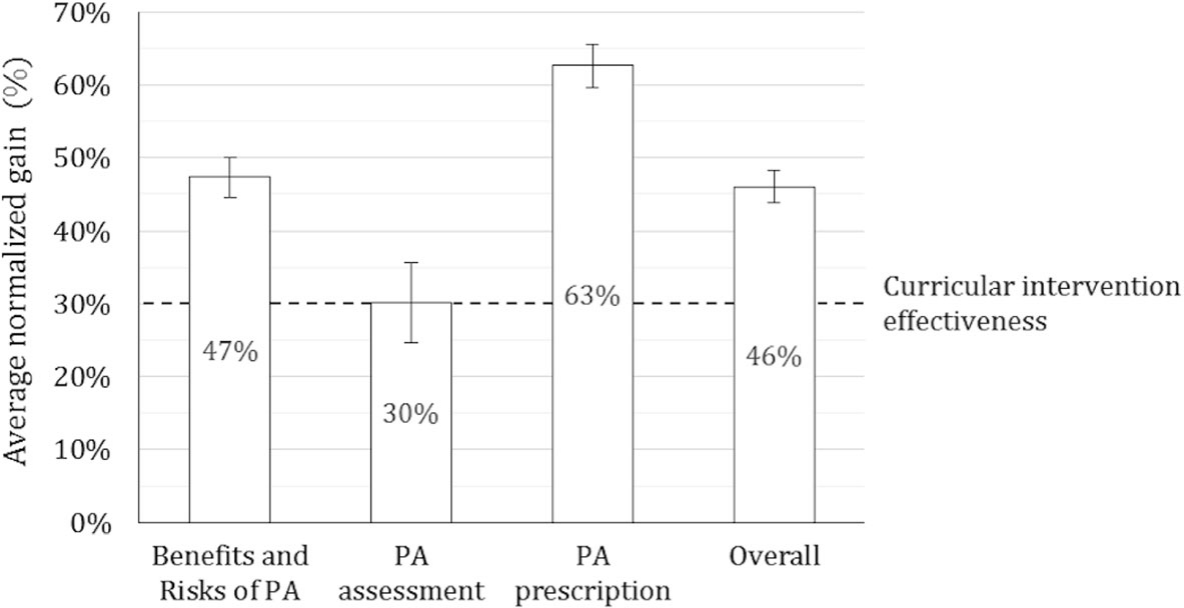
Class-average normalized gain ± standard error among all workshops (*n* = 41): Class-average normalized gain was calculated using the formula; Average actual gain/maximum possible gain (1- pre score). Class-average normalized gain was analyzed for each theoretical course topic. Class-average normalized gain at 30% or higher considers a curriculum to be effective [31]

Overall, class-average normalized gain was 46% (CI: 42–51%). The module with the highest normalized gain was PA prescription (63% CI 57–69%). The curricular intervention effectiveness for each topic, measured via normalized gain, was greater or equal than 30% for all of the course modules (Fig. 4).

Multivariate ordinary least squares regression analyses were completed. There were two models, one for the pre-test and one for the post-test. Factors that were found to be significantly (*p* < 0.001) associated with the pre-test score in the model including all specialties (*R* ^*2*^ = 0.22; *p* < 0.001) were age 40 years and older (β = −0.05), meeting aerobic PA recommendations (β = 0.03), and being obese or overweight (β = −0.04) and MD specialty except for GP and Cardiology (Table 3).

**Table 3:** Pre and Post-test multivariate ordinary least squares regression analyses

	Pre-test score model				Post-test score model			
	All specialties (*n* = 870)		Family medicine only (*n* = 74)		All specialties (*n* = 870)		General medicine only (*n* = 298)	
	*R* ^*2*^ = 0.22 (*p* < 0.001)		*R* ^*2*^ = 0.52 (*p* = 0.017)		*R* ^*2*^ = 0.20 (*p* < 0.001)		*R* ^*2*^ = 0.21 (*p* = 0.001)	
	Coefficient	*p*-value	Coefficient	*p*-value	Coefficient	*p*-value	Coefficient	*p*-value
Country (reference, Argentina)								
Bolivia	0.02	0.64			0.09	**0.01**		
Chile	0.13	**0.01**			0.15	**0.00**	0.17	**0.03**
Colombia	0.07	0.06	−0.23	**0.03**	0.14	**0.00**	0.16	**0.02**
Costa Rica	0.08	**0.03**	−0.21	0.18	0.11	**0.00**	0.11	0.11
Ecuador	0.00	0.92			0.03	0.39	−0.02	0.89
Mexico	0.04	0.33	−0.34	**0.00**	0.08	**0.02**	0.05	0.59
Nicaragua	0.08	0.06	0.08	0.64	0.04	0.24	−0.03	0.73
Puerto Rico	0.14	**0.02**			0.24	**0.00**	0.25	**0.01**
Dominican Republic	−0.05	0.20	−0.32	**0.01**	0.07	**0.03**	0.09	0.23
Uruguay	0.05	0.37	−0.17	0.15	0.15	**0.00**	0.14	0.09
Venezuela	0.06	0.25			0.14	**0.00**	0.23	0.09
Specialty (reference, Other specialties)								
General Practitioner	0.00	0.74			−0.01	0.49		
Family Medicine	0.06	**0.00**			0.02	0.27		
Internal Medicine	0.06	**0.00**			0.02	0.21		
IM Sub.	0.06	**0.01**			−0.01	0.68		
Pediatrics	0.07	**0.01**			0.01	0.75		
Cardiology	0.02	0.54			−0.01	0.80		
Sports Medicine	0.13	**0.00**			0.06	**0.00**		
Age > 40	−0.05	**0.00**	−0.07	**0.02**	−0.03	**0.00**	−0.06	**0.00**
Male sex	0.01	0.14	0.02	0.60	0.01	0.27	−0.01	0.36
Daily sitting time (hours), continuous	0.00	0.61	0.01	0.22	0.00	0.18	0.00	0.64
Perception of poor health status	−0.01	0.25	−0.09	**0.02**	−0.03	**0.02**	−0.04	**0.03**
Complies with aerobic PA recommendation	0.03	**0.01**	0.06	0.05	0.01	0.46	0.00	0.94
Complies with muscular PA recommendation	−0.01	0.32	−0.07	**0.02**	0.00	0.60	0.02	0.26
Evaluate PA in their clinical practice	0.00	0.96	−0.11	0.05	0.00	0.78	0.00	0.96
Recommends PA in their clinical practice	0.02	0.31	0.18	**0.01**	0.01	0.47	0.01	0.73
Overweight and obesity (BMI > 25 kg/m2)	−0.02	**0.04**	0.01	0.82	−0.01	0.34	0.00	0.76
Agree with: “Doctors are responsible for promoting adequate physical activity levels”	0.03	0.31	−0.17	0.05	0.03	0.21	0.03	0.52
Agree with: “I will be able to provide more credible and effective counseling if I stay fit”	−0.03	0.15	0.08	0.34	−0.01	0.66	0.01	0.84
Agree with: “I am effective in helping my patients to be physically active”	−0.01	0.18	0.01	0.86	−0.02	0.07	−0.02	0.16
Constant	0.61	**0.00**	0.96	**0.00**	0.70	**0.00**	0.69	**0.00**

*IM Sub* Internal medicine subspecialties others than Cardiology (immunology, endocrinology, pulmonology, nephrology, gastroenterology, hematology, intensive care specialists, and infectious diseases specialists) *p*-values <0.05 appear in bold

When stratified by specialty, the model for family medicine MDs explained a larger proportion of the variability in the pre-test scores (*R* ^2^ = 0.52; *p* < 0.017). Significant factors for this specific model were country where workshop was held (Colombia, Mexico, Dominican Republic; β = −0.23; −0.34 and −0.32, respectively), age 40 years and older (β = −0.07), perception of a poor health status (β = −0.09) as well as meeting the muscle-strengthening PA recommendation (β = −0.07) (Table 3).

The post-test model including all specialties explained a modest proportion of the variability (*R* ^*2*^ = 0.20; *p* < 0.001). Significant factors for this model were all countries where workshop was held except Ecuador, Sports Medicine specialty (β = 0.06), age 40 years and older (β = −0.03) and perception of a poor health status (β = −0.03). When stratified by specialty, the model for GPs explained a modest proportion of the variability (*R* ^2^ = 0.21; *p* = 0.001) Significant factors for this model were country where workshop was held (Chile, Colombia, Puerto Rico; β = 0.17; 0.16 and 0.25, respectively), age 40 years and older (β = −0.06), perception of a poor health status (β = −0.04) as well as meeting the muscle-strengthening PA recommendation (β = −0.07) (Table 3).

**Fig. 5:**
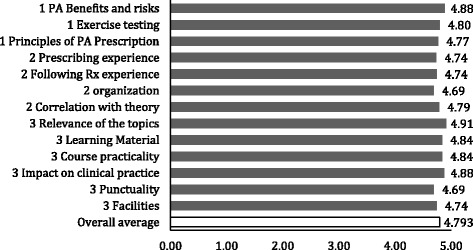
Overall course satisfaction: A survey was administered at the end of the course evaluating subjectively the course satisfaction within each section of the course. Five corresponds to the highest possible score and zero the lowest

Finally, the overall course satisfaction, as assessed via a self-reported evaluation at the end of the workshop was graded on average with 4.78/5 points. All specific components of the course were scored above 4.5/5 points, including the 3 course modules, relevance of the theoretical and practical components of the course, prescribing experience and course logistics among others (Fig. 5).

## Discussion

In this study we found that a one-day PA workshop can be an effective continued education strategy to improve MDs’ knowledge on exercise assessment, counseling and prescription. We used a pre-test/post-test quasi-experimental study, a common methodology in education research, to evaluate this educational strategy, implemented in a diverse population of MDs from 12 different countries in Latin America. We found the average normalized gain to be 46%, and according to previous studies, a class-average normalized gain of 30% or higher renders a curriculum to be considered effective [29]. Thus, in general our workshop curriculum was effective in generating the desired knowledge gain and particularly for the PA prescription module of the course. In addition, MDs that benefitted the most were PCPs (GPs and family medicine in particular), as shown by their higher relative knowledge gain (30% CI: 26–34% and 29% CI: 20–38% respectively). Furthermore, when stratifying the results by country of origin we found that Dominican Republic had the lowest pre-test scores and as a result the highest knowledge gain of all the countries. On the contrary, Chile had the highest pre-test score and one of lowest knowledge gains in addition to Nicaragua. Higher pre-test scores were found in Chile, Costa Rica and Dominican Republic, which indicates that education on PA and lifestyle medicine and preventive counseling for medical doctors may not be homogenous in the Latin American region.

Addressing physical inactivity constitutes one of the priority areas in the global health agenda to control the global burden of NCDs. Several evidence-based strategies to improve PA in the population are needed, from environmental interventions to integration of PA into health care settings. However, education on PA is not part of the medical curriculum in most medical schools in the world. This workshop provided participants with tools to properly assess patient’s PA levels, give brief behavioral counseling and prescribe exercise as part of treatment plans. There is evidence that brief advice to patients is a cost-effective approach to improve patient’s activity levels [34].

Similarities between countries were found regarding demographical characteristics, such as age, gender and BMI. Fifty-three percent of participants reported compliance with the international aerobic PA recommendations. This relatively high compliance could indicate that most participants may have had a particular interest in the workshop influenced by their personal PA behaviors. However, according to the National Survey of Nutrition of Colombia (ENSIN) 50% of Colombians meet the physical activity recommendations when taking into account their transportation method and recreational activities [35]. This is close to the prevalence found in our study of 53% compliance. In addition, there could have been an overestimation by the self-report nature of the assessment [36, 37]. In contrast, 51% of participating MDs were overweight or obese and the average sitting time was 5.6 ± 3.5 h/day, both of which are important independent risk factors for increased cardio metabolic risk and all-cause mortality [38]. In some previous studies, MDs have reported moderate to high physical inactivity rates, with similar prevalence of chronic disease and obesity as the overall population [39]. The most recent data in Colombia shows prevalence of abdominal obesity of 62% in women and 39,8% in men [35]. Interestingly, most MDs agreed that “if they stayed fit”, they would be capable of giving more credible and better PA counseling to patients. Furthermore, in the multivariate regression analysis we found that compliance with the PA recommendations was associated with a higher score in the pre-test, in particular for family medicine MDs. Therefore, physically active MDs appear to have more knowledge about exercise prescription. We and others have identified the strong link between personal PA behaviors and PA counseling practices [39–42]. The findings in the present study also seem to provide support to this theory driven by the fact that one of the main barriers MDs face is not feeling confident enough to give PA recommendations in an intellectual as well as a practical level. When asked about how often they were assessing patient’s PA levels and providing counseling, 73% reported they do it often or always; However it is important to take into account potential self-report overestimation, social-desirability and selection bias of the sample [36, 37].

When analyzing the results of the workshop by topics, it was evident that participants were in general aware of the benefits and risks of PA, and thus, they had a modest knowledge gain in this module (Table 2). However, the lack of baseline knowledge was more evident for the PA assessment and PA prescription modules of the workshop; This is in line with previous reports showing that MDs lack in-depth knowledge, tools and skills to effectively assess patient’s activity levels and carry out brief PA behavioral counseling and exercise prescriptions [43]. The question with the highest knowledge gain asked about specific tools for cardiovascular risk assessment before prescribing exercise, followed by questions related to the “5As” of counseling (Ask, Advise, Assess, Assist, and Arrange) and the correct recommendation for strength training in healthy adults. Accordingly, teaching MDs about screening and prescription using short lectures and practice-based workshops is likely to expand their knowledge on these topics. Consequently we saw a significant increase between the pre-test and post-test scores across specialties. There is a documented lack of lifestyle medicine as part of undergraduate medical schools’ plans. For example, Only 13% of 102 United States medical schools included PA and health in their curricula [40, 41]. As a result, short, one-day continued education workshops could become an effective strategy to address this problem in the short-term while larger curricular interventions are established in undergraduate MD training.

Previous studies have found that PCPs report lack of training and self-efficacy to support behavior change as important barriers to provide effective PA counseling [23]. Our course’s methodology addressed these barriers by providing background information on these topics using lectures and also by a practical workshop where MDs played the role of patients and had the opportunity to interact with colleagues that performed PA assessment and provided behavioral counseling and a written exercise prescription. A higher knowledge gain among PCPs is a critical component of the EIM® initiative to integrate PA-related care as a standard of care in clinical settings, as this particular group of MDs has the most impact in implementing preventive strategies and lifestyle medicine counseling for patients with NCDs or risk factors [43, 44]. The role of GPs implementing lifestyle medicine and preventive PA counseling in the LA region is critical since GPs account for 75% of the MD workforce and most patients do not have access to specialists, according to the Colombian GPs association [45]. In addition, specialties labeled as “other” had an even greater relative knowledge gain (44% CI 34–55%). This can be explained by the fact that these professionals rarely engage in preventive counseling as part of their daily practice. However, a core objective of the EIM initiative is to make “PA a vital sign for every patient, every visit” and certainly non-PCP and other specialties can reinforce the important message of PA for health [19]. Sports medicine specialists had the lowest relative knowledge gain (14%), as they are the most adept professionals in prescribing exercise and their background knowledge is largely focused on this area [46]. Nevertheless, we still observed significant improvements in post-test scores and qualitatively they valued the course. We believe sports medicine and others specialists can become leaders in helping train other colleagues in the basics of PA prescription [46]. To increase the potential impact of EIM educational efforts, we recently began to implement a train-the-trainer capacity building strategy were a number of sports medicine and other specialists become certified in our workshop and then help replicate it in the regions and health systems where they practice.

This study had several strengths. It is the first to propose a short-term continuing medical education regional strategy proven to be effective and measureable in filling the gap to train MDs on PA prescription in 12 different countries [26]. The short-term nature of the workshop was also a benefit since it diminished external factors that could have had an effect on the internal validity and facilitated replication and scale-up. Other continued medical education interventions have found short-term strategies to be an effective way to increase knowledge [47]. The study also had some weaknesses. Since the workshop was open to any interested MD, a volunteer self-selection bias is possible. Despite this, there was enough variability in terms of participants’ perceptions about their own PA habits and counseling practices, baseline PA knowledge and specialty. We only evaluated the immediate impact of the workshop on knowledge gain; therefore the impact of the workshop on actual clinical practice is unknown. Nevertheless, we are in the process of collecting follow-up data every 6 months to evaluate this. Finally, even though the test we used was not a standardized questionnaire, the questions were taken from the ACSM/EIM® resources and international PA guidelines, which are the gold-standard reference tools to train health care providers and fitness professionals in exercise prescription [29].

## Conclusion

This one-day workshop had a positive impact on the knowledge gain of participating MD’s on the topic of PA prescription. Although all groups of specialties increased knowledge, GPs and family medicine MDs benefitted the most. We found that baseline skills and knowledge gain is partially determined by the MDs’ area of specialty, country of practice, age, and to some extent, their personal PA habits. Hence, future workshops should focus on developing and implementing different strategies in order to address specific sub-groups of MDs and support their personal PA goals as an important correlate of their preventive counseling pactices. This short course can be an effective continuing education strategy for teaching PA assessment, counseling and prescription to MDs in Latin America, a topic rarely included in the training of MD’s in the region and the world. The short and long-term impact that a one-day workshop may have in the MDs clinical practice to assess, counsel, prescribe and refer patients based on their PA needs, remains to be evaluated.

## Additional file

Additional file 1: Table S1. Relative gain by country where the EIM workshop was offered. (DOCX 79 kb)

## Abbreviations

EIM®: Exercise is medicine®
GPs: General practitioners
HCPs: health care professionals
MD: Medical doctor
NCD’s: Non- communicable diseases
PA: Physical activity
PCP: Primary care physicians
