# Supplementary material for: One-day workshop-based training improves physical activity prescription knowledge in Latin American physicians: a pre-test post-test study
Source: BMC Public Health. 2016 Dec 5;16:1224. doi: 10.1186/s12889-016-3883-2 (PMC5139105; doi:10.1186/s12889-016-3883-2)
Supplement: Additional file 1: Table S1. — Relative gain by country where the EIM workshop was offered. (DOCX 79 kb) [file 12889_2016_3883_MOESM1_ESM.docx]

**Supplemental table S1: Relative gain by country where the EIM workshop was offered.**

|  |  |  | **Relative Gain** | | | | **Total pre-test score** | | **Total post-test score** | |
| --- | --- | --- | --- | --- | --- | --- | --- | --- | --- | --- |
| **Country** | **N** | **%** | **Mean** | **Std. Dev.** | **CI Lower Limit** | **CI Upper Limit** | **Mean** | **Std. Dev.** | **Mean** | **Std. Dev.** |
| Argentina | 38 | 3.64 | 15.0% | 32.2% | 4.4% | 25.6% | 63.8% | 14.2% | 71.2% | 16.5% |
| Bolivia | 29 | 2.78 | 26.5% | 25.5% | 16.8% | 36.2% | 64.0% | 13.7% | 78.6% | 12.7% |
| Chile | 23 | 2.2 | 13.4% | 14.8% | 7.0% | 19.8% | 80.0% | 14.5% | 88.9% | 9.4% |
| Colombia | 491 | 47.03 | 27.6% | 32.2% | 24.7% | 30.4% | 69.6% | 13.5% | 85.7% | 10.7% |
| Costa Rica | 83 | 7.95 | 22.2% | 35.4% | 14.5% | 30.0% | 69.3% | 13.3% | 81.4% | 10.2% |
| Ecuador | 26 | 2.49 | 25.5% | 46.3% | 6.8% | 44.2% | 62.5% | 14.2% | 74.4% | 11.9% |
| Mexico | 176 | 16.86 | 21.6% | 27.8% | 17.4% | 25.7% | 68.3% | 13.0% | 80.3% | 11.4% |
| Nicaragua | 26 | 2.49 | 9.3% | 17.1% | 2.4% | 16.2% | 67.7% | 14.2% | 72.9% | 14.1% |
| Puerto Rico | 9 | 0.86 | 27.6% | 23.9% | 9.3% | 45.9% | 77.8% | 13.0% | 96.7% | 5.6% |
| Dominican Republic | 104 | 9.96 | 72.5% | 129.9% | 47.2% | 97.8% | 53.8% | 18.6% | 76.2% | 11.9% |
| Uruguay | 25 | 2.39 | 31.3% | 25.2% | 21.0% | 41.7% | 65.2% | 12.9% | 83.2% | 10.3% |
| Venezuela | 14 | 1.34 | 18.3% | 20.2% | 6.6% | 29.9% | 69.6% | 11.7% | 81.4% | 14.1% |
|  |  |  |  |  |  |  |  |  |  |  |
| **TOTAL** | 1,044 | 100 | 29.3% | 52.5% | 26.1% | 32.4% | 67.4% | 14.9% | 82.2% | 12.2% |
